# Mechanisms and Research Progress of Phosphate-Solubilizing Microorganisms in Promoting Sustainable Crop Production

**DOI:** 10.3390/microorganisms14092022

**Published:** 2026-09-11

**Authors:** Junfang Wang, Xin Yu, Peiqun Dong, Yifan Li, Ying Zhang, Gang Wang

**Affiliations:** 1 School of Life Sciences, Henan University, Kaifeng 475004, China; wangjunfang@henu.edu.cn (J.W.);; 2 International School of Technology, Henan University, Kaifeng 475004, China

**Keywords:** phosphate-solubilizing microorganisms, phosphorus solubilization mechanisms, organic phosphorus mineralization, functional genes, biofertilizers

## Abstract

Phosphorus is an essential macronutrient for crop growth, but soil-available phosphorus is commonly lacking. Conventional chemical P fertilizers suffer from low utilization efficiency and dependence on finite phosphate rock reserves, necessitating the urgent development of green and efficient alternative strategies for phosphorus management. Phosphate-solubilizing microorganisms (PSMs) are capable of converting insoluble inorganic and organic phosphorus in soils into plant-available forms, thereby serving as key biological resources for enhancing phosphorus use efficiency, reducing dependence on chemical fertilizers, and promoting sustainable agricultural development. This review systematically covers the taxonomic diversity and multifaceted applications of PSMs, elucidates the mechanisms of inorganic P solubilization and organic P mineralization, and separately summarizes recent advances in functional genes involved in inorganic and organic P degradation. It further identifies key bottlenecks restricting PSM development and envisions the use of emerging technologies to transition PSM inoculants from empirical screening toward rationally designed precision deployment, thereby strengthening the scientific and technological foundation for enhancing phosphorus use efficiency and promoting green agricultural sustainability.

## 1. Introduction

Phosphorus (P) is an essential macronutrient for plants, and its bioavailability is a primary determinant of crop productivity [1]. At the cellular level, phosphorus serves as a structural component of adenosine diphosphate (ADP) and adenosine triphosphate (ATP), playing an irreplaceable role in energy transfer and storage [2]. It is also intrinsically involved in photosynthesis, respiration, membrane biogenesis, glycolysis, and enzymatic regulation [3], and constitutes an integral part of nucleotides, phospholipids, phosphoproteins, and coenzymes [4], thereby underpinning core life processes, including genetic expression, metabolic regulation, and structural organization. At the plant level, phosphorus promotes root development, enhances stem strength, improves grain formation, and boosts biological nitrogen fixation [5]; its adequate supply is therefore a prerequisite for the completion of the entire growth cycle and the achievement of high yields [6]. However, phosphorus availability in arid and semi-arid soils is generally low [7,8]. Phosphorus deficiency can lead to poor root development, impaired chlorophyll synthesis, delayed fruit setting, and reduced grain size, thereby severely decreasing both crop yield and quality [1]. In addition to directly supplementing crop phosphorus nutrition, phosphorus fertilization increases the availability of metal ions in the soil and enhances the nutritional stimulatory effects of root exudates, thereby increasing soil microbial diversity and abundance [9], which in turn indirectly improves rhizosphere nutrient mobilization. Collectively, phosphorus not only constitutes the material foundation for plant metabolic regulation and yield formation but also serves as a critical driver of rhizosphere ecological functions. Although phosphorus fertilization can effectively promote crop growth and enhance stress tolerance, excessive application, while boosting grain production, has also led to the accumulation of soil contaminants, structural degradation, and a decline in soil fertility, thereby constraining the sustainable development of agriculture [10]. Long-term nitrogen and phosphorus addition drives the plant community toward monopolization by a single functional group, accompanied by a sustained decline in the biomass share of other functional groups, ultimately resulting in reduced community species diversity [11]. At present, there is a continuing surplus of phosphorus in cropland soils, and the associated environmental risks are becoming increasingly conspicuous. Due to the heterogeneous distribution of soil phosphorus—with elevated levels in surface layers but deficiency of available phosphorus in deeper horizons—coupled with the rapid transformation of 75–90% of added phosphate fertilizers into forms that are not readily assimilable by plants via fixation, precipitation, and complexation, the use efficiency of applied phosphorus in the current crop cycle is exceptionally low: approximately 20% for grain crops and below 10% for vegetables and fruit trees [12,13]. Approximately 67% of global agricultural soils are affected by phosphorus limitation, which represents a critical bottleneck to improving crop productivity and efficiency [14,15]. In China, soil available phosphorus (AP) levels have demonstrated a general upward trajectory. Taking Guangxi as an illustrative case, surface AP concentrations increased substantially between 1984 and 2019: the proportion of land area with AP below 10 mg/kg declined from 80% to 3%, whereas that with AP in the range of 10–40 mg/kg rose to 97%, a shift primarily attributable to intensive phosphate fertilizer inputs. Nonetheless, the current-season phosphorus recovery efficiency in China stands at only 10–25% [16], which is markedly insufficient relative to crop demands. As a consequence, substantial phosphorus surpluses are transported into aquatic systems via surface runoff and leaching, intensifying the threats of non-point source pollution and waterbody eutrophication [17,18]. Adding to the gravity of the situation, phosphate fertilizer manufacturing is critically dependent on phosphate rock, which constitutes a non-renewable strategic mineral asset. The global reserves of phosphate rock are not only limited but also strikingly uneven in their geographic distribution, with approximately 70% or more confined to Morocco and Western Sahara, and the remaining share largely allocated to a handful of nations, including China, the United States, and Russia. Furthermore, high-grade, readily accessible phosphate deposits are progressively depleting, and the bulk of the remaining stocks are of low grade, which in turn drives a persistent escalation in extraction and beneficiation costs [19]. Notwithstanding its prominent position in global phosphate rock reserves, China is also grappling with diminishing ore grades, mounting extraction challenges, and intensifying environmental pressures [20]. The inherent scarcity of phosphate resources, coupled with their asymmetrical geopolitical distribution, has engendered recurrent price volatility and long-term supply uncertainties for phosphate fertilizers, thereby posing a direct threat to the stability of global food systems [21]. Consequently, an exclusive reliance on chemical phosphate fertilizers is inadequate to sustainably fulfill crop phosphorus requirements, underscoring the imperative to devise alternative strategies—including biofertilizers, phosphorus synergists, and precision fertilization practices—that can curtail over-dependence on chemical inputs, elevate phosphorus utilization efficiency, and safeguard soil health and microbial community integrity. Addressing these challenges is of paramount and urgent importance for ensuring food security, advancing the green transition of agriculture, and realizing ecological preservation in cropland systems.

Given the escalating prominence of environmental issues, phosphorus fertilization practices in crop cultivation have increasingly oriented toward reconciling crop yield objectives with ecological preservation, leading to the development of a soil phosphorus-based building-up and maintenance management approach [22]. Over recent decades, a substantial body of research has been devoted to phosphorus utilization in agricultural soils, encompassing the mechanisms of phosphorus immobilization, soil phosphorus speciation, approaches to enhancing crop phosphorus uptake efficiency, and the biological potential inherent in plant–microbe interactions to orchestrate efficient phosphorus acquisition and utilization by crops [23,24]. Among these lines of inquiry, plants have been shown to possess a suite of adaptive strategies for efficient phosphorus capture from the soil. Beyond modulating their own root architecture and physiological attributes, plants engage in symbiotic or associative interactions with PSMs, through which they capitalize on microbial activities to mobilize and take up soil phosphorus [25,26]. PSMs constitute a broadly distributed microbial cohort in natural ecosystems, with the capacity to convert recalcitrant soil phosphorus into soluble moieties, thus effectively ameliorating the pervasive issue of low phosphorus bioavailability. Through their solubilizing activities, otherwise immobile phosphorus fractions that are inaccessible to plants are transformed into plant-assimilable soluble forms, thereby elevating soil soluble phosphorus pools, diminishing the requisite input of chemical phosphate fertilizers, and attenuating the environmental perils engendered by their excessive application [27,28]. Consequently, as a pivotal nexus within the plant–soil–microbe continuum, PSMs exhibit substantial potential for augmenting soil phosphorus availability, reducing dependency on synthetic phosphorus inputs, and curtailing agricultural non-point source pollution. They have accordingly emerged as indispensable biological assets for the construction of ecologically sound and sustainable agroecosystems. Although the phosphorus-solubilizing function of phosphate-solubilizing microorganisms (PSMs) has been extensively studied, the impacts of their interactions with nitrogen, potassium, and micronutrients on overall plant nutrition remain poorly synthesized. In particular, critical knowledge gaps persist regarding the constraints of soil heterogeneity, the integration of multi-nutrient interactions, the understanding of ecological factor interplay, and the development of precision application frameworks. These unresolved issues represent the key gaps that this review aims to address. To this end, we systematically synthesize the diversity, multifunctional traits, phosphorus-solubilizing mechanisms, and key functional genes of PSMs, and critically analyze the major challenges facing their field application as well as future research directions. This review is intended to provide a theoretical reference for the efficient utilization of PSMs in sustainable agricultural systems.

## 2. Soil Phosphorus Forms

Phosphorus in soil is a key nutrient element essential for plant growth and development, and is also a major factor limiting plant growth and crop yield. Soil phosphorus occurs mainly in two forms—inorganic phosphorus and organic phosphorus [29].

### 2.1. Soil Organic Phosphorus

Organic phosphorus (Po) in soil is defined as the phosphorus fraction residing in humus and other organic constituents, which normally constitutes 30–65% of the total soil phosphorus pool. In forest soils characterized by high organic matter accumulation, the Po proportion can reach up to 90% of total phosphorus, thereby rendering it a significant source of plant-available phosphorus for numerous terrestrial ecosystems. Nevertheless, only approximately 1% of this organic fraction is directly assimilable by crops [30]. Soil organic phosphorus (Po) exhibits diverse forms and varying stability, and its sources mainly include biological metabolic processes (e.g., orthophosphate assimilation) and biological residues (e.g., microbial, animal, and plant debris), as well as synthetic compounds such as organophosphate pesticides, detergents, and flame retardants. According to its chemical structure, soil organic phosphorus can be classified into the following categories: (1) phosphate esters, such as phospholipids, sugar phosphates, and phytate [31]; (2) phosphoproteins, such as enolase, kinases, and casein; (3) phosphate anhydrides, such as acetyl phosphate and nucleotides (e.g., ADP, GDP, and ATP); (4) phosphonates, represented by the herbicide glyphosate; (5) phosphoramidates, including pesticides such as fenamiphos, parathion, and chlorpyrifos [32]; and (6) organic polyphosphonates, with typical representatives being the scale inhibitors ethylenediamine tetramethylenephosphonic acid and hydroxyethylidene diphosphonate [33]. These organic phosphorus compounds are relatively stable in soil; however, they can be transformed into inorganic phosphorus via microbial mineralization, thereby becoming accessible for plant assimilation [34].

### 2.2. Soil Inorganic Phosphorus

Soil inorganic phosphorus constitutes the primary phosphorus pool in soils, generally representing 50–90% of the total soil phosphorus content. On the basis of solubility and existing forms, soil inorganic phosphorus may be categorized into the following groups: (1) water-soluble phosphorus, which occurs predominantly in the soil solution as ionic species such as H_2_PO_4_^−^ and HPO_4_^2−^, and provides a phosphorus source that is directly assimilable by plants. However, its concentration in soil is typically low and is subject to the influence of multiple factors, including soil pH, the rate of phosphate fertilizer application, and the abundance and binding status of phosphorus in the soil solid phase [35]; (2) adsorbed phosphorus, which mainly refers to the phosphate ions (e.g., H_2_PO_4_^−^ and HPO_4_^2−^) that are adsorbed and fixed onto the surfaces of positively charged soil colloids. This fraction of phosphorus is generally low in content but can be taken up by plants under certain conditions [36,37]; and (3) mineral phosphorus, which is the predominant form of inorganic phosphorus in soil, accounts for approximately 99% of the total inorganic phosphorus pool. It mainly comprises calcium phosphates, iron phosphates, and aluminum phosphates [38,39,40]. These phosphate compounds differ in their solubility and consequently in their availability to plants. Generally, phosphorus is mainly immobilized as iron and aluminum phosphates in acidic soils, whereas in alkaline soils it primarily exists as calcium phosphates [41]. PSMs promote the transformation of sparingly soluble inorganic phosphorus through the secretion of protons, organic acids, and phosphatases, thereby facilitating phosphorus mobilization [42,43,44].

## 3. Diversity of Phosphate-Solubilizing Microorganisms

Currently, identified PSMs mainly include phosphate-solubilizing bacteria (PSB), phosphate-solubilizing fungi (PSF), and cyanobacteria [45,46,47]. Bacteria exhibiting phosphate-solubilizing activity are mostly affiliated with genera such as *Acinetobacter*, *Bacillus*, and *Rhizobium*, among others (Table 1). PSF are predominantly affiliated with genera including *Apophysomyces*, *Aspergillus*, and *Rhizophagus*, among others (Table 1). Among the entire population of PSMs, soil PSB represent 1–50% of the PSMs, whereas PSF comprise 0.1–0.5%, and the majority of these microorganisms are localized in the rhizosphere [48,49,50]. Soil physicochemical properties, fertilization regimes, crop species, and soil organic matter content are key factors influencing the species composition, abundance, and distribution of phosphate-solubilizing microorganisms (PSMs) [51]. To date, numerous PSMs have been isolated and screened from plant rhizosphere soils by a wide range of researchers. Multiple PSB strains that promote rice growth have been isolated from soil samples collected from various sources, including *Enterobacter asburiae* 30FPSSB1, *E. mori* NTTC11, *Priestia aryabhattai* KNB6, *P. aryabhattai* 49KNA2, *P. megaterium* 65KNA2, and *Bacillus* sp. 38DFWA [52]. A total of 35 PSMs isolated from the rhizosphere of willow were assigned to 12 genera, namely *Bacillus*, *Cedecea*, Cellulosimicrobium, *Delftia*, *Ensifer*, *Paenibacillus*, *Pantoea*, *Phyllobacterium*, *Pseudomonas*, *Rhizobium*, *Sinorhizobium*, and *Staphylococcus* [53]. Soil is a reservoir of diverse microbial communities, where PSMs are abundantly present. Their metabolic activities are critical for integrated nutrient management, as they both facilitate plant nutrient acquisition and act as biofertilizers that harmonize with ecological sustainability, ultimately boosting crop production.

## 4. Agricultural Applications of PSMs

PSMs exhibit multiple functions in agricultural systems, including plant growth promotion, environmental remediation, and biocontrol.

### 4.1. Plant Growth Promotion

The pathways by which PSMs promote plant growth include solubilizing insoluble phosphates, mineralizing organic phosphorus to enhance soil-available phosphorus supply, and secreting bioactive substances such as auxins to regulate plant metabolism.

#### 4.1.1. Nutrient Balance and Yield Increase

Multiple PSMs have been demonstrated to improve crop phosphorus nutrition and enhance yield through phosphate solubilization. For example, the PSM strains *Burkholderia* XQP35 and *Raoultella* SQP80 are capable of dissolving calcium phosphate and calcium phytate, thereby promoting the transformation of phosphorus fractions through participation in phosphorus cycling, increasing soil-available phosphorus content, and ultimately promoting maize growth [27]. Application of phosphate-solubilizing *Bacillus* spp. to common bean increased yield by 12% and reduced phosphate fertilizer application by 33% [84]. Similarly, rice seeds primed with PSMs optimized the rhizosphere microenvironment and nutrient uptake, thereby promoting rice growth [41]. These findings indicate that PSMs can be used to regulate plant phosphorus balance and overall development.

#### 4.1.2. Hormone-Mediated Growth Promotion

Certain PSMs have been reported to directly enhance plant growth and development via the production of phytohormones, including auxins, ethylene, and gibberellins [85]. It was demonstrated that five efficient PSMs isolated from the rhizosphere of legumes, with the ability to produce IAA and ACC deaminase, increased the biomass of faba bean and pea by 77.78–88.88% [86]. The phosphate-solubilizing bacteria *P. aeruginosa* AKRC7 and *E. coli* AKRC16 have been shown to produce phosphatases, phytase, and IAA, thereby promoting 100% germination of tomato seeds and markedly enhancing seedling phosphorus content and vigor index [87]. Similarly, *Pseudomonas corrugata* SP77, *P. koreensis* LT62, and *P. frederiksbergensis* G62 are capable of producing phytase, IAA, and siderophores, which significantly increase alfalfa dry weight and fresh nodule weight [88]. In addition, the ACC deaminase produced by certain PSMs has been shown to degrade the ethylene precursor ACC under adverse conditions, including drought and salinity, consequently curbing the excessive buildup of ethylene and mitigating its inhibitory effects on crop growth. Concurrently, IAA upregulates the expression of ACC synthase genes, thereby elevating ACC levels, while ACC deaminase catalyzes the conversion of surplus ACC into ammonia and α-ketobutyrate, providing a nitrogen source for PSMs and simultaneously preventing ethylene oversaturation. This concerted action enables PSMs to sustain low ethylene titers alongside adequate IAA accumulation under stressful environments, thus fostering plant development and reinforcing stress resilience [89,90,91]. The aforementioned PSMs, characterized by their multiple growth-promoting functions, are capable of mobilizing soil phosphorus via biomineralization, and are expected to serve as eco-friendly substitutes for chemical fertilizers.

### 4.2. Ecological Remediation by PSMs

Phosphate-solubilizing microorganisms (PSMs) are involved in environmental remediation of two main pollutant groups, namely heavy metals and organophosphate pesticides.

#### 4.2.1. Heavy Metal Remediation

Heavy metal pollution of soils constitutes a grave threat to both food security and human health. Consequently, the development of efficient strategies for the remediation of contaminated soils has emerged as a pressing environmental priority. PSM-assisted phytoextraction is one of the approaches to enhance the removal of toxic metals from contaminated soils. Studies have shown that inoculation with the Pb-resistant PSB strain *Leclercia adecarboxylata* L1-5 increased soil-available phosphorus content, increased shoot and root biomass of *Celosia cristata* by 34.90% and 55.56%, respectively, and increased Pb content in roots and shoots by 3.06-fold and 0.72-fold, respectively, significantly enhancing the plant’s Pb extraction capacity [92]. In addition, the exopolysaccharide (EPS)-producing PSB strain *E. soli*, isolated from a mangrove rhizosphere, has been shown to effectively remove Cr through biosorption [93]. Similarly, the marine-derived PSB strain *Curtobacterium luteum* exhibited adsorption capacity for multiple metals, with maximum removal rates of 61.32% for Cr, 54.68% for Cd, 43.88% for Zn, and 34.92% for Cu [94]. These results highlight the potential application value of phosphate-solubilizing bacteria in the remediation of various heavy metal contaminations.

#### 4.2.2. Organophosphate Pesticide Degradation

Organophosphorus compounds are highly toxic and recalcitrant to natural degradation, necessitating their effective removal from contaminated environments. Bioremediation, owing to its high efficiency and low cost, has emerged as a key strategy for addressing such pollution. In applied research on PSMs, multiple strains have been demonstrated to possess organophosphorus-degrading capability. For example, the PSB strain *Klebsiella* sp. can effectively degrade the pesticide triazophos [95]. The strain *Brucella intermedia* Msd2 possesses both monocrotophos-degrading activity and multiple plant growth-promoting traits, including ammonia production, enzyme production, phosphate solubilization, zinc solubilization, potassium solubilization, and ACC deaminase activity, thereby demonstrating dual functionality in pollution remediation and plant growth promotion [96]. Furthermore, genetic engineering has introduced an innovative strategy to this research area—the heterologous expression of the organophosphorus hydrolase gene *opd* from *Flavobacterium* in *Saccharomyces cerevisiae* has been achieved, enabling both the detection and degradation of the insecticide paraoxon [97]. Phosphate-solubilizing microorganisms (PSMs) can not only enhance plant phosphorus utilization, but also show significant application potential in the bioremediation of organophosphorus-contaminated environments.

### 4.3. Biocontrol

Phosphate-solubilizing microorganisms (PSMs) can inhibit plant pathogens through mechanisms such as competitive colonization, secretion of enzymes and allelochemicals, and induced resistance. For example, the phosphate-solubilizing strains *Pseudomonas moraviensis*, *B. cereus*, and *B. aryabhattai* produce HCN, cellulase, and chitinase, which not only significantly inhibit *Fusarium oxysporum* and *Verticillium dahliae* but also promote the growth of leguminous plants [93]. It has been demonstrated that the PSB *P. taiwanensis* WJP-7 enhanced cotton yield by 19.51% and achieved 50.53% disease control against Verticillium wilt [98]. Furthermore, the phosphate-solubilizing strains *Brevibacterium frigoritolerans*, *Bacillus valismortis*, and *Streptomyces venezuelae* showed potent antagonistic effects against *Fusarium oxysporum* and *Botrytis cinerea* [99]. Collectively, PSMs possessing biocontrol properties hold promise as environmentally sustainable substitutes for chemical fertilizers and pesticides.

## 5. Phosphorus Solubilization Mechanisms

**Figure 1:**
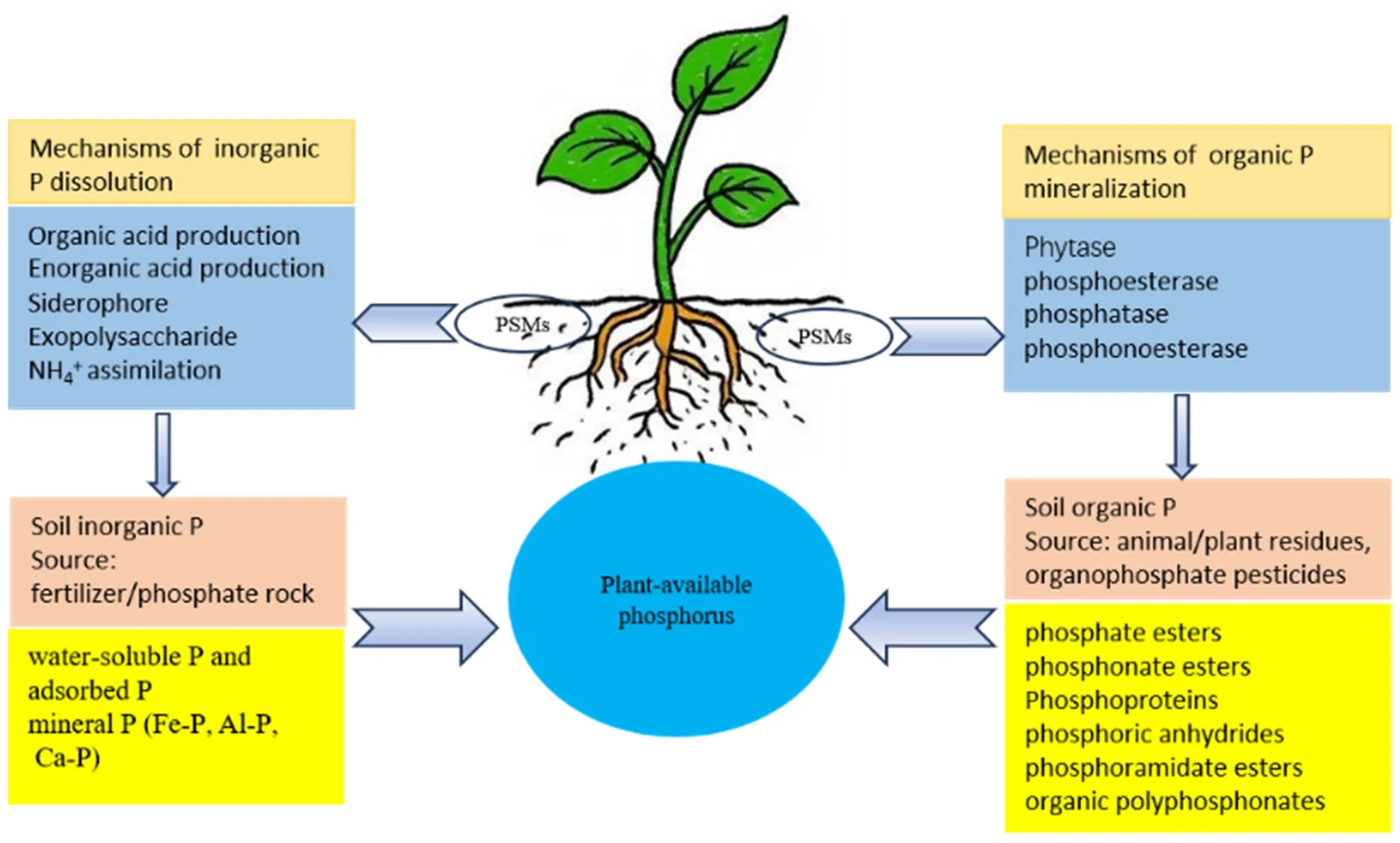
Schematic diagram illustrating the mechanisms of inorganic phosphate solubilization and organic phosphorus mineralization in soil by phosphate-solubilizing microorganisms. P: phosphorus; Fe-P: ferric phosphate; Al-P: aluminum phosphate; Ca-P: calcium phosphate.

To make effective use of PSMs for increasing crop productivity, it is imperative to elucidate the mechanisms by which they solubilize sparingly soluble phosphates, even though these mechanisms remain incompletely understood. Available evidence suggests that PSMs transform unavailable soil phosphorus into plant-available phosphorus via diverse routes. According to the types of substrates, these mechanisms may be classified into two categories: (1) the dissolution of inorganic phosphorus (Pi) via acidification, protonation, chelation, and complexation; and (2) the mineralization of organic phosphorus (Po) through the action of phosphatases, phytase, and C-P lyase. The concerted action of these two mechanisms enhances the soil’s phosphorus-supplying capacity and consequently promotes plant growth (Figure 1).

### 5.1. Mechanisms of Inorganic Phosphate Solubilization

In soil, the dissolution of sparingly soluble inorganic phosphates such as Fe-P, Al-P, and Ca-P is mainly achieved through the following pathways:

#### 5.1.1. Organic Acid Secretion

During growth, PSMs secrete low-molecular-weight organic acids, such as gluconic acid, citric acid, fumaric acid, lactic acid, propionic acid, and ascorbic acid, among others (Table 2). These organic acids facilitate the dissolution of inorganic phosphates mainly through the following mechanisms: (1) Acidification. By releasing protons, organic acids lower the local pH, thereby directly dissolving sparingly soluble phosphates (e.g., calcium phosphate and iron phosphate) [100,101]. Under low pH conditions, phosphate is primarily present as the monovalent anion H_2_PO_4_^−^, which represents the most plant-available form. With increasing pH, this monovalent species is progressively transformed into the sparingly soluble divalent (HPO_4_^2−^) and trivalent (PO_4_^3−^) species. Consequently, the secretion of organic acids facilitates the conversion of divalent and trivalent phosphate species into soluble monovalent forms via acidification (i.e., pH reduction). For example, the inorganic PSF *Penicillium oxalicum* PSF-4 can dissolve tricalcium phosphate and iron phosphate, with the former solubilization mainly involving the production of oxalic acid, malic acid, and formic acid, while the latter primarily involves lactic acid, gluconic acid, and citric acid. Control experiments demonstrated that phosphorus solubilization could be achieved by the addition of hydrogen ions alone, indicating that the solubility product of the phosphorus source, the composition of carboxylic acids, and solution pH are key factors influencing the dissolution efficiency of different phosphorus sources [102]. (2) Chelation. Low-molecular-weight organic acids (e.g., gluconic acid, oxalic acid, and citric acid) chelate Fe^3+^, Al^3+^, and Ca^2+^ bound to phosphates via their hydroxyl and carboxyl groups, disrupting the mineral structure and thereby releasing PO_4_^3−^ [103,104]. For instance, gluconic acid produced by phosphate-solubilizing *Pseudomonas* spp. can be further oxidized to 2-keto-D-gluconic acid and related derivatives. These compounds, via the combined effects of acidification and metal-ion chelation, destabilize the crystal lattice of insoluble phosphates and consequently liberate soluble phosphate species [105,106]. (3) Ligand exchange. Organic acid anions facilitate the desorption of phosphate from soil matrices, such as amorphous aluminum oxide and kaolinite, by replacing surface-bound PO_4_^3−^ via ligand exchange mechanisms [107,108]. (4) Synergistic metabolic actions. Organic acid production is frequently coupled with microbial respiration (which generates CO_2_, leading to carbonic acid formation), thereby lowering pH and chelating metal ions to improve phosphorus availability. Moreover, PSMs can reduce rhizosphere pH through gaseous exchange and proton–bicarbonate equilibrium, thus facilitating the release of bound phosphate [109]. Through the synergistic interplay of acidification, chelation, ligand exchange, and co-metabolic processes, PSMs efficiently convert recalcitrant inorganic phosphates in soil into forms that are directly accessible to plants.

#### 5.1.2. NH_4_^+^ Assimilation-Driven Proton Extrusion

Multiple lines of evidence indicate that NH_4_^+^ assimilation is a common mechanism contributing to inorganic phosphate solubilization by PSMs. Microorganisms release protons (H^+^) through ammonium assimilation or nitrification, leading to a decrease in rhizosphere pH, which in turn promotes the hydrolysis of Fe/Al-bound phosphates and consequently increases the concentration of soluble phosphorus in the soil [118]. Specifically, the phosphate-solubilizing capability of *Pantoea agglomerans* MMB051 has been demonstrated to be not only dependent on organic acid production but also correlated with active cell growth, wherein proton release associated with NH_4_^+^ assimilation plays a pivotal role, while the presence of NO_3_^−^ as the nitrogen source suppresses its solubilizing activity [119]. Research has revealed that the phosphate-solubilizing mechanism of *Advenella kashmirensis* DF12 is not exclusive; the concentration of solubilized phosphorus was not directly correlated with gluconic acid levels but was significantly positively correlated with proton concentration. Proton efflux associated with NH_4_^+^ assimilation was identified as a key factor in phosphate dissolution [120]. Collectively, these findings indicate that NH_4_^+^ assimilation universally contributes to inorganic phosphate solubilization by PSMs. Accordingly, the application of ammonium-based nitrogen fertilizers in phosphorus-deficient soils is advisable, as it can concurrently supply nitrogen, improve phosphorus efficiency, and support the goals of reducing fertilizer input while enhancing crop productivity.

#### 5.1.3. Exopolysaccharide Production

Bacterial extracellular polymeric substances (EPS), as a major constituent of biofilms, are primarily composed of proteins, polysaccharides, nucleic acids, and peptidoglycan, and are characterized by resistance to acid, alkali, salt, and heat, as well as good miscibility [121]. It has been demonstrated that EPS produced by the phosphate-solubilizing strain *Rhizobium tropici* can form strong complexes with phosphates, thereby facilitating the transformation of residual and calcium-bound phosphorus into water-soluble fractions, and consequently improving phosphorus bioavailability [122]. Additional research has revealed that the strain *Enterobacter* EnHy-401, characterized by high production of EPS and organic acids, exhibits potent phosphate-solubilizing activity. While EPS alone is incapable of solubilizing tricalcium phosphate, it exerts a synergistic effect with organic acids on phosphate dissolution, and the extent of this synergism is contingent upon both the source and concentration of EPS, with the homologous EPS demonstrating the greatest efficacy. Additional research has revealed that *Enterobacter* EnHy-401, characterized by high production of EPS and organic acids, exhibits potent phosphate-solubilizing activity. While EPS alone is incapable of solubilizing tricalcium phosphate, it exerts a synergistic effect with organic acids on phosphate dissolution, and the extent of this synergism is contingent upon both the source and concentration of EPS, with the homologous EPS demonstrating the greatest efficacy. By retaining free phosphorus, EPS shifts the solubilization equilibrium toward dissolution, suggesting that phosphorus retention constitutes another important mechanism underlying phosphate solubilization [123]. Beyond functioning as a structural matrix of biofilms that bolsters the resistance of bacterial communities to harsh environments, EPS also plays an essential role in soil phosphorus cycling.

#### 5.1.4. Siderophore Production

Siderophores are low-molecular-weight secondary metabolites with iron-chelating activity, produced by microorganisms or plants in response to iron limitation. Through high-affinity chelation of Fe^3+^, they form ferric-siderophore complexes that are translocated into cells via specific membrane receptors, accompanied by the concomitant release of phosphate (PO_4_^3−^), thus mediating the solubilization of phosphates [124]. The phosphate-solubilizing actinobacterium *Streptomyces* CoT10 exhibits high efficiency in mobilizing phosphorus sources and produces a broad spectrum of siderophores. It was demonstrated for the first time that its iron-phosphorus solubilizing function is directly mediated by siderophores. Upon application to oil tea (*Camellia oleifera*), this strain facilitates phosphorus fraction transformation through the mobilization of insoluble iron and phosphorus in the soil, leading to a 15.35% increase in available phosphorus content, and consequently promoting plant growth as well as phosphorus and iron acquisition. Collectively, these results provide a novel strategy for phosphorus mobilization in iron-rich soils [125]. It has been demonstrated that *Pseudomonas* sp. TC952 achieves inorganic phosphate solubilization via a dual mechanism involving acidification and chelation, mediated by the secretion of organic acids and siderophores [126]. In addition to alleviating iron stress in microorganisms, siderophores also serve as a key strategy for phosphate-solubilizing microorganisms to mobilize recalcitrant soil phosphorus, functioning either in concert with organic acids or through independent pathways.

### 5.2. Mechanisms of Organic Phosphorus Mineralization

Organic phosphorus (Po) accounts for approximately 30–50% of total soil phosphorus, and its mineralization depends on multiple hydrolytic enzymes secreted by PSMs and plant roots [125]. Based on pH optima, phosphatases are classified into acid phosphatases (ACP), predominantly derived from plants and fungi, and alkaline phosphatases (ALP), primarily of microbial origin [127]. According to substrate specificity, these enzymes fall into two categories: non-specific phosphohydrolases, such as non-specific acid phosphatases (NSAPs) and ALP, which are mainly secreted by microorganisms and are capable of hydrolyzing a broad range of organic phosphate esters including nucleotides and sugar phosphates [128]; and specific phosphohydrolases, such as phytase that specifically degrades phytate—the most abundant but poorly available organic phosphorus pool in soil—and C-P lyase that acts on carbon–phosphorus bonds [127]. The concerted action of these two classes of enzymes progressively mineralizes organic phosphorus into orthophosphate (H_2_PO_4_^−^ and HPO_4_^2−^), which is subsequently available for plant uptake (Figure 1).

#### 5.2.1. Non-Specific Phosphatases

Microorganisms secrete non-specific phosphatases (also termed phosphomonoesterases), which mainly include non-specific acid phosphatases (NSAPs) and ALP. Both are capable of hydrolyzing a broad spectrum of organic phosphate esters, such as nucleotides and sugar phosphates, yet differ markedly in their pH optima and molecular properties [129]. NSAPs are key enzymes involved in the mineralization of organic phosphorus by PSMs, facilitating the release of inorganic phosphate [128]. Based on sequence homology and cellular localization, NSAPs are classified into three categories: class A and class B are soluble periplasmic proteins with pH optima of 5.5–6.0 and 6.0–6.5, respectively, and are predominantly found in the Enterobacteriaceae; class C comprises membrane-bound lipoproteins with acidic pH optima, which are more commonly distributed in non-enterobacterial species [128]. All three classes are mesophilic enzymes (temperature optima of 30–37 °C, active range of 20–45 °C), yet exhibit distinct thermostability profiles. They also differ markedly in substrate preference, cellular localization, and evolutionary origin. Notably, a single bacterial strain can simultaneously express multiple classes of NSAPs, suggesting functional complementarity and cooperative participation in organic phosphorus mineralization [130]. ALPs also play a significant role in organic phosphorus mineralization and are primarily secreted by microorganisms. Their biochemical properties vary considerably among bacterial species: pH optima generally range from 8.0 to 10.0 (e.g., pH 8.5 for lactic acid bacteria and pH 9.5 for *Bacillus* spp.), whereas the ALP of an Antarctic psychrotolerant *Bacillus* strain exhibits an optimal pH of only 7.0 [131]. Temperature adaptability is also notably diverse: mesophilic bacteria exhibit temperature optima of 37–55 °C, thermophiles can reach 60–70 °C, while psychrophiles display optima around 25 °C with poor thermostability (complete inactivation within 50 min at 60 °C) [127]. Furthermore, ALP is widely distributed across different soil types and functions in concert with NSAPs [132], collectively driving the mineralization and turnover of organic phosphorus. Studies have demonstrated that *Gongronella* sp. w5 recruits PSB through the hydrolysis of plant-derived sucrose, which releases fructose. In the tripartite interaction involving the plant, strain w5, and PSB, w5 not only contributes a portion of the alkaline phosphatase (ALP) activity but also stimulates PSB to secrete ALP, thus facilitating the release of soil-available phosphorus and promoting the growth of Medicago truncatula [133]. An additional study has demonstrated that in acidic soils, both plant species and rhizosphere effects modulate the abundance, community structure, and functional activity of bacteria involved in the secretion of acid and alkaline phosphatases. Notably, acid phosphatase activity was found to be more pronounced and to play a pivotal role in the mineralization of organic phosphorus [134]. NSAPs exert a significant function in microbially mediated organic phosphorus mineralization, thereby effectively enhancing soil phosphorus bioavailability and promoting plant growth.

#### 5.2.2. Phytase

Phytase catalyzes the stepwise hydrolysis of phosphate groups from phytate (myo-inositol hexakisphosphate), releasing myo-inositol and inorganic phosphate (Pi). Based on the initial site of dephosphorylation, phytases are classified into 3-, 6-, and 5-phytases [135]. According to catalytic mechanisms, they are divided into four families: histidine acid phosphatases (HAPs), commonly found in Enterobacteriaceae and fungi; β-propeller phytases (BPPs), predominantly derived from *Bacillus* spp. and characterized by high stability; cysteine phytases (CPs), present only in rumen microorganisms; and purple acid phosphatases (PAPs), widely distributed in fungi and bacteria [135]. The mineralization pathway follows a sequential dephosphorylation order: phytate → inositol pentaphosphate → inositol tetraphosphate → inositol triphosphate → inositol diphosphate → inositol 2-monophosphate (2-IP) [136]. The pH and temperature optima of phytases vary with source: bacterial, fungal, and yeast phytases exhibit pH optima of 2.7–8.0, 2.5–6.0, and 2.5–5.5, and temperature optima of 37–75 °C, 40–70 °C, and 40–77 °C, respectively [137]. The high-phytase-producing strain *Pseudomonas* sp. S3-10 has been demonstrated to significantly enhance soybean growth, with concomitant increases of 388.15% in soil phytase activity and 365.05% in rhizosphere-available phosphorus concentration [138]. Phytate, as a major component of soil organic phosphorus, can be mineralized by microbial phytase to release plant-available phosphorus, thereby enhancing phytate utilization efficiency, reducing the need for phosphate fertilizer inputs, and mitigating the risk of phosphorus loss to water bodies [139]. Similarly, the application of three bacterial strains with elevated phytase activity—*Serratia* sp. C2, *Serratia marcescens* N4, and *Citrobacter* sp. S10—to barley led to marked improvements in chlorophyll content, plant height, root development, and phosphate accumulation in both leaves and roots relative to the control [140]. Collectively, by liberating available phosphorus from otherwise inaccessible phytic acid complexes in soil, phytase demonstrates considerable promise for enhancing crop productivity and nutritional quality.

#### 5.2.3. Phosphonatases

Phosphonatase, also referred to as C-P lyase, catalyzes the cleavage of C-P bonds and is a critical enzyme class involved in the degradation of organophosphonates by certain phosphate-solubilizing microorganisms [141]. The microbial degradation of glyphosate occurs through two principal routes: the aminomethylphosphonic acid (AMPA) pathway and the C-P lyase pathway. *Stenotrophomonas maltophilia* GP 1 was the first strain demonstrated to utilize the C-P lyase pathway for glyphosate degradation [142]. The phosphate-solubilizing strain *Comamonas odontotermitis* P2 can utilize glyphosate as its sole carbon and/or phosphorus source for growth, with a degradation efficiency of up to 90%. This strain possesses both glyphosate oxidoreductase (GOX) and C-P lyase, suggesting that it may degrade glyphosate via the coordinated action of these two pathways, thereby exhibiting considerable potential for bioremediation [142]. Collectively, phosphonatase, through its cleavage of C-P bonds, serves as a key player in the microbial degradation of organophosphonates.

#### 5.2.4. Phosphoesterases and Their Substrate Spectra

Organophosphorus pesticide residues are resistant to natural degradation and thus represent a sustained hazard to ecosystems. Phosphatases derived from microorganisms, notably organophosphorus hydrolase (OPH), can hydrolyze organophosphate compounds, thereby attenuating their toxicity by several orders of magnitude [143]. OPH mediates the cleavage of ester bonds, generating low-toxicity products that are amenable to further microbial metabolism. For instance, the OPH enzyme from *Flavobacterium* sp. ATCC 27551 facilitates the conversion of paraoxon to p-nitrophenol (PNP) and diethyl phosphate (DEP). OPH is a Zn^2+^-dependent enzyme with a broad substrate range, capable of hydrolyzing diverse organophosphorus compounds, including phosphotriesters, phosphorothioates, fluorophosphates, and parathion [143]. It has been demonstrated that mixed bacterial populations of *Pseudomonas* sp. and *Flavobacterium* sp. produce OPH and utilize methyl parathion and parathion as sole carbon sources for growth, wherein the former hydrolyzes these compounds to p-nitrophenol, which is subsequently degraded to nitrite by the latter [144]. Recombinant *Escherichia coli* expressing the OPH-encoding gene, when coupled with immobilization techniques, has been shown to accelerate the degradation of organophosphorus neurotoxins, including chlorpyrifos [145]. Additionally, the methyl parathion hydrolase (MPH) produced by the phosphate-solubilizing strain *Plesiomonas* sp. M6 can hydrolyze methyl parathion [146]. Moreover, the cytochrome P450 enzyme from *Phanerochaete chrysosporium* has been reported to degrade triphenyl phosphate (TPhP) at a rate exceeding 60% [147]. In addition to the aforementioned enzymes, diisopropyl fluorophosphatase (DFPase) is a calcium-dependent phosphotriesterase capable of specifically cleaving phosphorus-fluorine (P-F) bonds [148]. As an example, the organophosphate acid anhydrolase (OPAA) originating from *Alteromonas* sp. HM01 demonstrates considerable catalytic activity toward the hydrolysis of organophosphorus compounds containing P-F bonds, including the nerve agent sarin and soman [149]. Collectively, phosphatases and organophosphorus hydrolases—such as OPH, PTE, MPH, and DFPase—serve a pivotal function in the degradation and detoxification of organophosphorus contaminants through the catalytic cleavage of P-O, P-F, and related bonds, thus offering effective degradation routes for both environmental cleanup and the treatment of neurotoxic agents.

The mechanistic basis underlying the ability of PSMs to “orchestrate” efficient phosphorus acquisition in plants lies in the formation of a signal-driven, multi-member collaborative dynamic regulatory network among plants, microorganisms, and the environment [150]. At the plant–microbe signaling interface, phosphorus deficiency triggers the active secretion of specific signal molecules by plant roots to recruit phosphate-solubilizing functional microbes [151]. Under phosphorus-deficient conditions, crop root exudates induce *pqqE* gene expression and *pqq* promoter activity in *Serratia* sp. S119 [152]. In terms of plant–microbe signaling, crops mediate the secretion of oxalic and citric acids through the GmSTOP1-3 transcription factor, which specifically enriches three groups of phosphate-solubilizing bacteria—Gammaproteobacteria, KF-JG30-C25, and Solirubrobacterales—whose abundance is significantly positively correlated with rhizosphere organic acid concentrations and the abundance of phosphorus cycling-related genes [151]. In *Arabidopsis thaliana*, the FERONIA receptor kinase has been shown to regulate root metabolite composition, thereby promoting the enrichment of phosphate-solubilizing rhizobacteria in response to phosphorus deficiency [153]. Furthermore, low-molecular-weight organic acids (LMWOAs) secreted by roots significantly increase the diversity and abundance of microbial communities harboring the *pqqC* and *phoD* genes, which in turn influences the expression levels of root phosphate transporters and acid phosphatases in crops [154]. At the level of multi-strain synergy, different functional groups of PSMs achieve collective effects that transcend the performance of individual strains through metabolic complementarity. Co-culture of *Aspergillus niger* and *Burkholderia cepacea* exhibits a significant synergistic effect on the solubilization of CaHPO_4_ [155]. Moreover, multi-omics studies have revealed that genes involved in accessory mechanisms and those encoding core phosphate-solubilizing functions are co-regulated by the same phosphorus starvation signaling pathway, forming an integrated regulatory network [38]. Furthermore, PSMs can establish tripartite interactions with arbuscular mycorrhizal (AM) fungi: the extraradical hyphae of AM fungi transport solubilized phosphorus to plant roots, thereby compensating for the poor mobility of phosphorus in soil [156]. The essence of how PSM “orchestrate” plant phosphorus acquisition lies in a multi-layered synergistic system in which plants establish “recruitment signals” through root exudates, different functional groups of PSMs members cooperate through division of labor, and symbiotic microorganisms such as AM fungi participate in phosphorus transport—a system that is orchestrated by plants, executed by microorganisms, and modulated by environmental conditions [154]. This conceptual framework marks a paradigm shift in our understanding of PSM functionality, from a focus on the phosphorus-solubilizing capacity of individual strains toward network-based regulation involving multiple members. Rather than passively relying on the solubilizing activity of a single microbial species, plants actively shape a functionally complementary and hierarchically structured microbial cooperative network through signal regulation and niche construction to achieve efficient phosphorus acquisition [152]. Elucidating the organizational principles and regulatory hubs of this network will provide a theoretical foundation for the precise manipulation of rhizosphere microbiomes and the optimization of PSM inoculant formulation strategies.

## 6. Key Phosphorus Cycling Functional Genes

PSMs play a pivotal role in soil phosphorus cycling through a dual mechanism: they solubilize insoluble inorganic phosphorus via the secretion of organic acids (e.g., gluconic acid), and mineralize organic phosphorus through the release of phosphatases and other enzymes, thereby modulating plant phosphorus availability [157]. The phosphorus cycling function of PSMs is regulated by a suite of key genes, which, according to the metabolic processes involved, fall into two categories: inorganic phosphorus solubilization-related genes and organic phosphorus mineralization-related genes.

### 6.1. Genes Involved in Inorganic Phosphorus Solubilization

The production and secretion of organic acids represent the principal mechanism underlying inorganic phosphate solubilization by PSMs, yet the molecular mechanisms governing acidolysis remain poorly understood. To date, only a limited number of genes implicated in phosphate solubilization have been identified, including *gad*, *gdh*, and *gcd* (all of which are involved in gluconic acid metabolism), *eno* (which encodes enolase), *ylil* (encoding aldose dehydrogenase), and the ABC transporter system gene *hlyB*, which is associated with organic acid secretion [158,159,160]. It has been demonstrated that the periplasmic glucose oxidation pathway of the PSB *Pseudomonas fluorescens* proceeds via the sequential action of glucose dehydrogenase (encoded by *gad*) and gluconate dehydrogenase (encoded by *gcd*), which catalyze the stepwise conversion of glucose to gluconic acid and subsequently to 2-ketogluconic acid [161]. It has also been demonstrated that *eno*, the gene encoding enolase in the inorganic phosphate-solubilizing strain *Burkholderia cenocepacia* 71-2, is involved in organic acid metabolism and has been implicated in phosphate solubilization [162]. Furthermore, multiple additional genes associated with organic acid metabolism have been characterized, including *bI-1* (encoding a BI-1 protein involved in acetate translocation), *poxB* (encoding pyruvate dehydrogenase), *gltA* (encoding citrate synthase), *ldh* (encoding lactate dehydrogenase), *gyaR* (encoding glyoxylate reductase), and *ghrB* (encoding glyoxylate/hydroxypyruvate/6-ketogluconate reductase), all of which are potentially involved in the phosphate solubilization process of PSMs [151,163,164,165]. Among the multitude of genes implicated in phosphate solubilization, the *gdh* gene and the *pqq* gene cluster have drawn particular interest [158]. The former encodes glucose dehydrogenase (GDH), which catalyzes the periplasmic oxidation of glucose to gluconic acid, while the latter is responsible for the biosynthesis of PQQ, an essential cofactor for GDH. Together, these genetic elements are involved in the gluconic acid metabolic pathway of Gram-negative bacteria [166]. The biosynthesis of pyrroloquinoline quinone (PQQ) is regulated by the *pqq* operon, which comprises five core genes—*pqqA*, *pqqB*, *pqqC*, *pqqD*, and *pqqE*. Among these, *pqqA* encodes the peptide precursor for PQQ biosynthesis, *pqqB* is potentially involved in the transmembrane transport of PQQ [163], and *pqqC* catalyzes the final step of PQQ synthesis. Consequently, microorganisms carrying the *pqqC* gene are frequently employed as model organisms in studies investigating inorganic phosphorus mobilization in soil, with higher *pqqC* abundance indicating greater phosphorus-solubilizing potential [166]. Furthermore, the abundance of *pqqC*-harboring microorganisms has been shown to correlate significantly with soil total phosphorus, total nitrogen, total potassium, organic carbon, available phosphorus content, and phosphatase activity, underscoring *pqqC* as a critical functional marker gene for assessing both organic phosphorus hydrolysis and inorganic phosphorus solubilization potential in soil [167]. A positive correlation exists between the integrity of the *pqq* gene cluster and phosphate-solubilizing efficiency. Notably, *Burkholderia cepacia* 51-Y1415, harboring the complete *pqqABCDE* gene cluster, demonstrated a phosphate-solubilizing activity exceeding 20-fold that of the cluster-defective strain; furthermore, the expression of this gene cluster is induced by low-phosphorus stress, thereby forming a positive feedback loop [168]. Beyond the *gcd* gene and the *pqq* system, certain inorganic PSMs are equipped with additional phosphate transport systems, including *pit* and *pstSCAB*. The *pit* gene is responsible for the transport of inorganic phosphate (H_2_PO_4_^−^/HPO_4_^2−^) under high-phosphorus conditions, while the *pst* system is induced under phosphorus-limiting conditions [169,170]. Collectively, these genetic components constitute a coordinated network that couples phosphate solubilization with phosphorus acquisition, thereby facilitating bacterial adaptation to fluctuating environmental conditions.

Many organic acids, including citric acid, α-ketoglutarate, succinic acid, and malic acid, are synthesized via the tricarboxylic acid (TCA) cycle, suggesting that genes associated with this pathway may play a role in the acidolysis-mediated regulation of inorganic phosphate solubilization by PSMs. This notion is supported by the observation that in three phosphate-solubilizing strains (HA2, HPA5, and KA1), the expression of genes involved in central metabolic routes—namely the ABC transport system, the TCA cycle, and glycolysis—was synergistically upregulated concurrent with phosphate solubilization [171]. Additional evidence has demonstrated that the expression of TCA cycle-related genes, including those encoding citrate synthase and isocitrate dehydrogenase, is markedly upregulated in media supplemented with insoluble phosphorus [41]. Nevertheless, the specific involvement of the TCA cycle in the acidolysis mechanism of phosphate-solubilizing microorganisms remains poorly understood and thus merits further exploration.

### 6.2. Genes Involved in Organic Phosphorus Mineralization

Phosphate-solubilizing microorganisms release orthophosphate (Pi), which is available for plant uptake, by hydrolyzing or phosphorylating organic phosphorus molecules through the secretion of various extracellular enzymes. The genetic basis of this process is governed by a suite of enzyme-encoding genes, primarily including phytase genes (*appA*, *phy*), phosphatase genes (*phoN*, *phoD*), and phosphoesterase genes (*phnX*).

#### 6.2.1. Phytase Genes

Microbial phytase genes are predominantly represented by the *appA* and phy gene families, which constitute the core genetic elements enabling phosphate-solubilizing microorganisms to release phosphorus from phytate via hydrolysis. The *appA* gene encodes an acid-preferring phytase and is ubiquitously present in Gram-negative bacteria, including *E. coli*, *Shigella*, and *Citrobacter* [172,173,174,175]. The *phy* gene encodes phytases with neutral to alkaline pH optima and is predominantly associated with genera including *Bacillus* and *Aspergillus* [176,177]. The contrasting distribution patterns and complementary biochemical attributes of the two gene families confer upon microorganisms the capacity to mineralize phytate across a broad pH spectrum, thereby positioning them as key functional genes governing soil phosphorus turnover.

#### 6.2.2. Phosphatase Genes

Soil phosphatases are mainly classified into two categories: alkaline phosphatase (ALP) and acid phosphatase (ACP) [129]. ALP is a non-specific phosphomonoesterase secreted by microorganisms, which drives organic phosphorus mineralization by catalyzing the hydrolysis of C–O–P bonds, and is primarily encoded by *phoA*, *phoD*, and *phoX* [178,179]. These three genes exhibit distinct niche differentiation: *phoA* is predominantly found in the periplasmic space of Gram-negative bacteria, where it hydrolyzes small-molecule organic phosphorus compounds transported from the extracellular environment [180,181]; *phoX* is mainly distributed in aquatic environments [51]; whereas *phoD* is widely present in both aquatic and terrestrial environments, occurring in actinomycetes and various Gram-positive and Gram-negative bacteria [182,183]. Among them, *phoD* expression is induced by phosphorus starvation and activated through the PhoR/PhoB two-component system, and its abundance has been recognized as a core bioindicator for assessing microbial phosphorus stress and mineralization potential in soils [184,185,186]. In addition, the *phoC* gene encodes an acid phosphatase with an acidic to neutral pH optimum. It also has a relatively broad distribution, occurring not only in various bacteria (e.g., *Morganella morganii*, *Zymomonas mobilis*, and *Streptomyces* spp.) but also in eukaryotes such as *Saccharomyces cerevisiae* [187,188,189]. In summary, the differential distribution and regulatory features of these phosphatase-encoding genes provide important targeted indicators for identifying functional microbial groups involved in soil phosphorus transformation and for evaluating mineralization potential at the molecular level.

#### 6.2.3. Functional Genes for Organophosphorus Pesticide Degradation

Unlike the mineralization genes discussed above, organophosphorus pesticide-degrading genes encode specialized hydrolases that disrupt the toxic moieties of pesticide molecules [190]. Such genes are predominantly situated on mobile genetic elements and are propagated through horizontal gene transfer, constituting essential genetic resources for bioremediation. The organophosphorus hydrolase gene *opd* remains the most thoroughly characterized and serves as a paradigmatic example [191]. It was initially discovered in the parathion-degrading strains *Pseudomonas diminuta* and *Flavobacterium* sp. Moreover, a specific region on the *opd*-bearing plasmid pPDL2 from *Flavobacterium* sp. was found to share an identical restriction endonuclease digestion pattern with the counterpart region on the *opd* plasmid pCMS1 from *P. diminuta* [192]. The *opd* gene is flanked, respectively, by ISF1sp1 (which encodes the complete *istAB* operon and belongs to the IS21 family) and a Tn3-like element (carrying t*npA*/*tnpR*, encoding transposase and resolvase) [193]. Divergently oriented relative to *opd*, ORF243 encodes a 27 kDa polypeptide implicated in the catabolism of p-nitrophenol, the primary breakdown product of parathion and methyl parathion. Furthermore, a 2.5 kb region upstream of *opd* harbors two ORFs that are co-directionally transcribed and exhibit high homology to *istA/istB*, acting synergistically to facilitate gene mobilization [143]. Given that the *opd* gene is frequently situated on mobile genetic elements, it exhibits considerable potential for spread within environmental microbial populations, and thus represents a paradigmatic case of rapid microbial adaptation to pesticide contamination stress [194].

Beyond the broad-spectrum *opd* gene, a number of degradation genes with high substrate specificity for certain pesticides have also been identified. For instance, the *mpd* gene from the recombinant strain *E. coli* BL21 encodes methyl parathion hydrolase (MPH), which catalyzes the conversion of methyl parathion to p-nitrophenol (PNP) [195]. The *hocA* gene from *Pseudomonas monteilii* encodes a novel phosphotriesterase that can efficiently degrade coroxon and exhibits broad substrate specificity toward both oxon-type and thion-type organophosphorus pesticides. This was the first time that an essential gene involved in organophosphorus degradation had been isolated from an organism capable of growing with organophosphorus pesticides as the sole phosphorus source [196]. It has been demonstrated that the *ophc2* gene from *Pseudomonas pseudoalcaligenes* encodes an organophosphorus hydrolase capable of efficiently hydrolyzing the organophosphorus insecticide ethyl parathion [197]. The *pde* gene from *Rhizobium* encodes a phosphodiesterase that catalyzes the cleavage of the phosphodiester bond in bis(2-chloroethyl) phosphate (BCEP), yielding a phosphomonoester [198]. Furthermore, the *phn* gene cluster in *E. coli* encodes C-P lyase, which is involved in the degradation of phosphonate compounds, including glyphosate, with concomitant release of inorganic phosphate [199]. The organophosphorus pesticide-degrading genes, represented by *opd*, encode specific hydrolases that precisely degrade pesticide molecules. They serve as important genetic resources for microbial bioremediation and hold significant value in green remediation and ecological safety.

## 7. Challenges and Future Perspectives of PSMs

### 7.1. Challenges of Phosphate-Solubilizing Microorganisms

Despite significant advances in research on PSMs, their large-scale field application and industrialization still face multiple challenges, which are mainly summarized as follows: (1) A notable scarcity of elite functional strains persists, and the underlying mechanisms governing colonization as well as the functional genes involved remain to be further elucidated. In field settings, introduced strains frequently encounter difficulties in establishing robust colonization, primarily owing to competition with native microbial communities and fluctuations in ambient factors, including temperature, pH, and water availability [160,200,201]. Moreover, a common challenge is the attenuation of strain efficacy when moving from controlled laboratory settings to field environments. The metabolic regulatory networks and functional genes governing phosphate solubilization in PSMs—including those linked to the TCA cycle—remain to be comprehensively elucidated [157,202]. The above constraints are primarily analyzed from the following aspects: (i) difficulty in determining mechanisms under field conditions. PSMs are considered to promote plant phosphorus uptake through both indirect and direct mechanisms. Indirect mechanisms involve the modulation of root morphology and resource allocation via phytohormone regulation, thereby enhancing phosphorus acquisition [200]. Direct mechanisms depend on the secretion of organic acids, protons, and siderophores, which lower pH or chelate metal ions to release insoluble phosphorus; some strains also produce enzymes that catalyze phosphorus solubilization [160]. While these pathways are readily detectable under laboratory conditions, they are difficult to distinguish from plant intrinsic effects in the field, and thus the actual mechanisms underlying rhizobacterial growth promotion remain elusive [203]; (ii) field performance of PSMs is significantly constrained by soil heterogeneity. In terms of soil texture, sandy soils generally favor PSM phosphorus-solubilizing activity due to their good aeration, whereas clay soils often compromise their phosphorus-enhancing effects because of compact structure, poor aeration, and strong phosphorus adsorption [204,205]. Soil organic matter content also exerts a dual influence: high organic matter may provide nutrients for PSMs, yet it can reduce the colonization activity of exogenous strains through competitive exclusion by native microorganisms; in low-organic-matter soils, PSMs face less competitive pressure but suffer from insufficient substrates for growth [206]. Moreover, high-organic-matter soils present a “double-edged sword” effect for the application of phosphate-solubilizing bacteria. While abundant organic matter supplies nutrients for microbial growth, it is often accompanied by increased diversity and abundance of native microbial communities, which may in turn intensify competitive exclusion against exogenous phosphate-solubilizing bacteria, thereby impairing their colonization capacity and functional performance in soil [204]. Mineral composition is equally critical—in alkaline soils, phosphorus is predominantly present as calcium phosphate, whereas in acidic soils, it occurs mainly as iron and aluminum phosphates [203]. Different strains exhibit marked variation in their capacity to solubilize phosphorus bound to different minerals [207,208]. Moreover, the commonly used readily soluble phosphorus sources in laboratory settings, such as tricalcium phosphate, cannot represent the complex phosphorus forms found in field soils, leading to an overestimation of the actual performance of isolates in laboratory screenings [209]; and (iii) microbial competition and antagonism represent a major challenge for the field application of PSMs [206]. Soil is a microecosystem characterized by intense competition. Once introduced, exogenous phosphate-solubilizing bacteria must compete with diverse and abundant native microbial communities for limited resources, including carbon sources, nitrogen sources, spatial niches, and root colonization sites—a competition that is particularly acute in field environments where carbon sources and energy are generally limited [210]. A study found that the growth-promoting effects of phosphate-solubilizing rhizobacteria on crops do not depend on soil phosphorus availability, but are significantly influenced by interstrain competition and interactions (including parasitic facilitation) [205]. Moreover, the beneficial effects of individual strains often cannot be sustained in mixed inoculation, suggesting that phosphate solubilization per se is not the direct mechanism underlying growth promotion, but may instead indicate the mutualistic potential of the strains [210]. In the construction of composite microbial inoculants, growth inhibition relationships among functional strains may compromise synergistic effects: the rhizosphere phosphate-solubilizing strain P1108 inhibits the growth of the potassium-solubilizing strain K2116, and the nitrogen-fixing strain N2126 also exhibits antagonism against K2116. When the three are mixed, P1108 becomes overwhelmingly dominant, accounting for 89.2% of the total cell count, while the colonization niches of the nitrogen-fixing and potassium-solubilizing strains are severely compressed [211]—the dominant strain weakens the colonization and functional expression of the weaker strains through resource competition or metabolic inhibition, resulting in the overall performance of the composite inoculant falling below expectations. Microbial competition and interactions are key determinants of the inoculation efficacy of phosphate-solubilizing bacteria, and their influence may even outweigh phosphorus availability per se [203]. In terms of the underlying mechanisms, resource competition primarily manifests as the struggle for nutrients and spatial niches, directly affecting the survival and functional expression of phosphate-solubilizing bacteria. Antagonism, on the other hand, is reflected in the direct inhibition or exclusion of exogenous strains by native microorganisms through the secretion of antibiotics and other antimicrobial substances [207]. For instance, it has been reported that antibiotic-producing soil bacteria can significantly reduce the phosphate-solubilizing activity of an introduced *Bacillus aryabhattai* strain [212]. Intense competition among even the most beneficial microorganisms leads to a decline in their abundance in soil, thereby diminishing their plant growth-promoting effects.

(2) The mechanisms underlying multi-strain synergy remain largely unclear. In natural environments, PSMs do not exist in isolation but form complex interaction networks with other rhizosphere microorganisms, such as nitrogen-fixing bacteria, arbuscular mycorrhizal fungi (AMF), and biocontrol agents [213,214]. However, the current understanding of the molecular mechanisms and ecological principles governing synergistic effects among multiple strains is still limited. Consequently, the formulation of composite microbial inoculants largely relies on empirical screening—often based on crop yield, biomass, or disease control efficacy—and lacks systematic theoretical guidance [215,216]. The above constraints are primarily analyzed from the following aspects: (i) research on PSM functionality has been overly phosphorus-centric, with insufficient attention to interactions with N, K, and micronutrients (Fe, Zn, Mn). PSMs synergize with nitrogen-fixers to enhance symbiotic N fixation; K-solubilizing strains release mineral K; and siderophores and organic acids chelate and solubilize micronutrients [217]. Co-culture of *Azospirillum formosense* with *Bacillus* spp. increases nitrogenase activity by 89% and P solubilization by 236% [218]; combined PSB+KSB inoculation improves soil P/K availability and crop nutrient uptake [219]; organic acids released during P solubilization also mobilize Fe and Zn, with some strains exhibiting both multi-mineral solubilization and siderophore production [220]; and co-inoculation with mycorrhizal fungi allows hyphae to transport solubilized P to roots, overcoming P immobility [221]. Thus, PSM growth-promoting efficacy depends on synergistic compatibility with other functional microbes and nutrients, requiring systematic optimization of strain combinations and application strategies [222]. (ii) Antagonistic relationships between PSM activity and nutrients also manifest at the level of plant chemical antagonism. In terms of plant nutrition, the mobilization of phosphorus by PSMs may indirectly affect the uptake of other nutrients. For example, excessively high available phosphorus in soil can suppress Zn uptake by plants (P–Zn antagonism), and the substantial release of phosphorus may consequently reduce the utilization efficiency of Fe and Zn in crops [222]. Moreover, excess potassium and phosphorus both interfere with nitrogen uptake in crops, while high nitrogen availability is also detrimental to potassium absorption [223]. The antagonistic effects at both the microbial interaction and plant chemical levels indicate that transitioning PSMs from “single-nutrient phosphorus regulation” to “multi-nutrient synergy” presents a complex theoretical challenge. Concerted efforts are required in strain functional compatibility screening, nutrient interaction network elucidation, and precision field application strategies to enhance phosphorus benefits while mitigating the risk of secondary nutrient deficiencies. (iii) In real field agroecosystems, PSM activity forms a multi-factor interaction network with nitrogen availability, oxidation-reduction potential (ORP), and heavy metal stress, collectively regulating phosphorus turnover. Ammonium nitrogen, which lowers rhizosphere pH through proton (H^+^) release from roots, is more favorable for enhancing phosphatase activity than nitrate nitrogen [224]. High nitrogen levels can enhance phosphatase pathways and alter soil available phosphorus (Olsen-P) [225]. Under low-phosphorus stress, inoculation with Bacillus increases nitrogen uptake in soybean by 94–248% [219], indicating a positive feedback loop of “phosphorus-promoted nitrogen enhancement” between PSMs and nitrogen-fixing bacteria. When ORP drops below 0 mV, chemical reduction becomes the dominant mechanism driving inorganic phosphorus dissolution, while PSMs are primarily involved in organic phosphorus transformation [226]. Under waterlogged conditions, biochar application can reduce ORP and enhance the diversity of phosphate-solubilizing bacteria, thereby coupling chemical reduction with biological activation [227]. Under anaerobic conditions, PSMs shift their energy metabolism from oxidative phosphorylation to substrate-level phosphorylation, accompanied by alterations in the profiles of organic acid secretion, which in turn affects phosphate-solubilizing efficiency [228]. Heavy metals exert a significant negative impact on PSMs, with their abundance decreasing as heavy metal concentrations increase. Among microbial taxa, Acidobacteria, Gemmatimonadetes, and Proteobacteria are sensitive to Pb and Zn, while Nitrospirae is susceptible to Cr, Cu, Pb, Zn, As, and Cd [229,230]. PSMs can immobilize heavy metals through multiple mechanisms, including phosphate-solubilizing precipitation, enzymatic transformation, and biofilm formation [231,232]. However, PSMs also exhibit mobilization effects—for instance, *Enterobacter* spp. can increase available phosphorus by 12.1–32.2% while simultaneously increasing available Cd by 43.4–104.3%, a property that facilitates Cd phytoextraction and remediation [233]. Nitrogen availability, ORP, and heavy metal stress often co-occur and interact synergistically: N application alters rhizosphere pH and DOM characteristics, thereby affecting heavy metal speciation and PSM activity; while water-induced ORP changes also influence heavy metal valence and toxicity. Understanding these interactions is theoretically important for precision P-cycling regulation, optimized biofertilizer application, and contaminated soil remediation.

(3) The stability of field performance is often unsatisfactory. PSM-based inoculants commonly suffer from limited shelf life and rapid loss of viable cells. Furthermore, the application methods required for these bioinoculants are substantially different from those used for traditional chemical fertilizers, thereby constraining both grower adoption and the potential for widespread deployment [210,234,235,236]. Poor field performance stability is a core bottleneck constraining the widespread adoption of PSM-based inoculants. The underlying causes can be analyzed from three perspectives: product characteristics, application technology, and farmer acceptance. (i) Unstable field performance. Although multiple physiological effects can collectively promote plant growth in controlled experiments, the expression of phosphorus-solubilizing mechanisms is often hindered under complex field conditions, making the outcomes difficult to predict [226,237]. In phosphorus-deficient soils, fungal inoculants generally outperform bacterial ones [220], whereas PSMs can also exert growth-promoting effects in high-nutrient environments, further increasing the complexity of application scenarios [238]. (ii) Insufficient shelf life and stability. Liquid inoculants typically have a shelf life of only 3–6 months at ambient temperature, while powdered formulations undergo rapid functional decline upon moisture absorption. Both storage and transport temperature/humidity fluctuations and the loss of functional genes contribute to shortening their effective lifespan [239]. Strain degeneration. Beneficial traits such as phosphate-solubilizing capacity are prone to mutation or loss during successive subculturing or long-term storage [240,241]. (iii) Disconnection in application technology. Refined application methods such as seed coating and root dipping are often incompatible with the coarse fertilization practices commonly used by farmers. The low adoption rate of specialized equipment, restricted application windows, the need to stagger application with fungicide use, and high labor and technical requirements collectively pose significant barriers [242]. Insufficient economic feasibility. The procurement cost of inoculants, combined with their inconsistent performance, makes it difficult to establish a stable benefit–cost ratio, leading to declining farmer confidence [243]. Moreover, technical and economic challenges in industrial production—including medium optimization and activity preservation—further reinforce these constraints. Together, these factors form a negative feedback loop: “unstable efficacy → shrinking demand → insufficient R&D investment → slow product improvement.” Breaking this cycle requires coordinated efforts in three areas: innovation in carrier materials, standardization of application practices, and verifiability of field efficacy.

(4) Fermentation production processes lag behind. Currently, most enterprises still adopt traditional fermentation approaches, which commonly result in problems such as rapid decline in strain viability, long fermentation cycles, and low viable cell yields [244,245]. There is an urgent need to introduce advanced technologies such as high-density fermentation and intelligent precise control to ensure product quality.

### 7.2. Perspectives of Phosphate-Solubilizing Microorganisms

To effectively address the above challenges, future research should focus on the following key directions: (1) Screening for novel and efficient functional strains. Multi-omics technologies, such as metagenomics and single-cell sequencing, should be integrated to discover low-abundance strains with high phosphate-solubilizing activity and their functional genes from natural environments. In addition, synthetic microbial consortia (SynComs) strategies should be employed to enhance rhizosphere colonization success and functional synergy [38,246]. Priority should be given to candidate strains that exhibit high safety, strong colonization ability, efficient interactions with crops and nutrients, multiple functional traits, and cost-effective production characteristics [247]. Conventional plate clearing zone assays and liquid culture methods are characterized by low throughput, long turnaround times, and poor predictive ability for field performance. In contrast, molecular screening based on functional genes offers a new avenue. Key genes such as *gcd* (encoding glucose dehydrogenase), *pqqABCDEF* (involved in PQQ cofactor synthesis), and *phoA*/*phoD*/*appA* (mediating organic phosphorus mineralization) are under the global regulation of the PhoR-PhoB two-component system [248]. These genes can be rapidly assessed via PCR amplification or metagenomic mining to evaluate the genetic phosphorus-solubilizing potential of individual strains or microbial communities [168]. However, genotype does not necessarily equate to field phenotype, necessitating the establishment of a multi-tiered validation framework linking “genotype–phenotype–field performance” [249]. To address the bottlenecks of low colonization success and competition from native microbial communities for exogenous strains, the synthetic microbial consortia (SynComs) strategy has been adopted to enhance rhizosphere colonization and growth-promoting stability through functional complementarity and ecological synergy [250]. Previous studies have demonstrated that designed synthetic consortia can coordinately achieve efficient phosphorus solubilization, phytohormone production, and growth promotion—for example, by enhancing nutrient uptake in wheat [251], and the EcoBiome consortium comprising *Erwinia rhapontici*, *Pseudomonas yamanorum*, and *Plantibacter* spp. has also been validated to exert synergistic effects under drought stress [252]. This strategy overcomes the functional limitations of single strains and offers new insights for the design of microbial consortia in high-efficiency phosphate-solubilizing biofertilizers. Future efforts should focus on optimizing community assembly logic and environmental compatibility to facilitate field-scale applications.

(2) Fermentation and composite formulation processes should be optimized. By implementing precise pH regulation, optimized feeding regimes, and temperature control, it is possible to realize high-density cell cultivation and enhanced production of secondary metabolites—including organic acids and phosphatases—thus guaranteeing the stability of product quality [245,253].

(3) Leveraging artificial intelligence and synthetic biology to advance PSM research. Targeted strategies—including gene editing, synthetic microbial community assembly, and interaction modulation—should be adopted to systematically improve the phosphate-solubilizing efficiency and environmental robustness of PSMs. AI-driven approaches should be utilized to construct big data models for strain–soil compatibility and to design structured synthetic consortia with high functional synergy. Furthermore, high-throughput screening platforms, such as machine vision systems, should be incorporated to expedite the transition from laboratory findings to scalable field applications [254,255,256]. The convergence of artificial intelligence and synthetic biology is propelling the development of phosphate-solubilizing microbial inoculants toward a new era of intelligent design, moving beyond traditional empirical screening. This integration provides revolutionary technological solutions for enhancing phosphorus use efficiency and advancing agricultural sustainability. The field performance of PSMs is constrained by multiple interacting factors, including soil heterogeneity (texture, organic matter, and mineral composition), competition from native microbial communities, the trade-off between plant carbon investment and phosphorus returns, and multi-nutrient interactions (N, K, Fe, Zn, etc.). The conventional linear approach of “single strain–single trait” is inadequate for addressing such a multi-dimensional nonlinear system [211,257]. Artificial intelligence (AI), particularly machine learning and deep learning algorithms, offers a powerful tool for integrating soil physicochemical parameters, climatic data, crop varieties, and microbiome information to establish optimal matching models between PSM strains and specific environments, thereby enabling precision deployment of phosphate-solubilizing bacteria [258,259]. Concurrently, synthetic biology can overcome the functional limitations of native strains—by assembling multi-gene modules and optimizing regulatory elements, it becomes possible to construct “smart strains” with integrated multifunctionality, including efficient phosphorus solubilization, nitrogen fixation, potassium mobilization, and siderophore secretion [260]. Furthermore, environmentally responsive genetic circuits can be designed to activate phosphorus-solubilizing gene expression only under low-phosphorus stress or specific rhizosphere signals, thereby avoiding unnecessary carbon costs and competitive burdens, and achieving “on-demand phosphorus solubilization” [261].

Artificial intelligence (AI) offers data-driven predictive and decision-making capabilities, while synthetic biology provides engineering tools for precision strain construction and functional optimization. Their roles are primarily manifested in the following aspects: (i) AI-driven strain screening and functional prediction. Traditional strain screening relies on empirical methods such as plate clearing zone assays, which are characterized by low throughput and high subjectivity. Machine learning models can efficiently decipher the non-linear relationships between environmental factors and phosphorus-solubilizing capacity [262]. For example, the Random Forest (RF) model performed best in predicting the phosphorus-solubilizing performance of Talaromyces funiculosus, with the highest solubilization achieved under the combination of pH 4.5 and calcium phytate [263]. In addition, the IGLOO machine vision system based on YOLOv8 can automatically detect colony clearance zones, achieving an accuracy of >90% and a relative error of <6%, while substantially shortening analysis time and eliminating subjective bias [257]. These cases demonstrate that AI has shown significant potential to replace traditional approaches in functional prediction and high-throughput screening. (ii) Synthetic biology-driven strain performance optimization. This approach encompasses three main strategies: targeted optimization of key phosphorus-solubilizing genes (*gcd*, *pqqE*, *phyA*) and their regulatory pathways via gene editing; design of structurally stable synthetic consortia based on principles of functional complementarity and niche differentiation [264]; and engineering of genetic circuits to endow strains with “on-demand” responsiveness [261]. In parallel, AI-driven promoter prediction and programmable synthetic circuit design are emerging as powerful tools for spatiotemporal regulation of gene expression. The synergistic application of these technologies is steering the development of composite microbial inoculants from empirical combination toward rational design. (iii) Data-driven precision application decision-making. Inoculation methods, densities, and environmental matching strategies are shifting from experience-based judgment to AI-assisted intelligent decision-making [265,266]. Localized application has been shown to significantly improve seedling growth parameters compared with uniform application, while the “seed + soil” dual inoculation strategy can effectively promote colonization due to its buffering effect. By integrating soil sensors, meteorological data, and crop models, AI-based decision systems can recommend optimal inoculation methods and parameters [265,267]. Response surface methodology (RSM) and artificial neural networks (ANN) are commonly employed to optimize inoculation density, with ANN demonstrating superior fitting capability for the phosphorus-solubilizing conditions of Pseudomonas sp. compared with traditional RSM [254]. The integration of synthetic biology tools—such as auxotrophy-based design and kill switches—with AI-driven soil diagnostics holds promise for achieving spatiotemporally precise release of microbial strains.

Despite the promising prospects of the abovementioned technological approaches, their translation from conceptual proposals to field implementation remains constrained by several critical shortcomings, including limited data availability, insufficient model generalization, and inadequate infrastructure [268,269]. The predictive capacity of AI models is highly dependent on the scale and representativeness of training data. Current agricultural microbiome data suffer from notable deficiencies in both standardization and diversity coverage, and small-sample laboratory data are often insufficient to generalize to complex field scenarios [270]. The complexity of rhizosphere interactions and their environmental dependence further constrain the transferability of these models [271]. More importantly, the relationship between model prediction accuracy and the quality of application recommendations is not linear—high-accuracy yield predictions do not necessarily translate into optimal input recommendations [268]. The high cost of sensors, poor data interoperability, and the “black-box” nature of deep learning models collectively undermine the scalability of these technologies and erode user trust [267,270]. Ultimately, models address the predictive question of “what will happen,” whereas application decisions must respond to the normative question of “what should be done,” requiring multi-objective trade-offs among economic costs, operational feasibility, and environmental risks [271]. Future breakthroughs will depend on the establishment of standardized datasets, long-term field validation systems, and the development of interpretable decision-making frameworks.

## 8. Conclusions

Phosphate-solubilizing microorganisms (PSMs) are core drivers of soil phosphorus cycling and vital biological resources for advancing the green transformation of agriculture. Their taxa encompass bacteria, fungi, actinomycetes, and arbuscular mycorrhizal fungi, which widely colonize the rhizosphere. They solubilize inorganic phosphorus through organic acid secretion, proton release, extracellular polymeric substance retention, and siderophore-mediated chelation, while mineralizing organic phosphorus via the synergistic actions of non-specific phosphatases, phytases, and C-P lyases, thereby converting insoluble soil phosphorus into plant-available forms. In parallel, PSMs directly promote plant growth by secreting indole-3-acetic acid (IAA), ACC deaminase, and siderophores; produce hydrolases and antimicrobial substances to suppress soil-borne diseases; and also exhibit ecological remediation functions, including heavy metal adsorption/immobilization and degradation of organophosphorus pesticides, thus demonstrating multifaceted synergistic application potential. Their phosphorus cycling function is coordinately regulated by a multi-layered gene network encompassing *pqq*-*gcd*, *phoD*, *appA*, *phy*, *phnX*, *opd*, and others, which constitutes the complete genetic foundation spanning from phosphorus activation and solubilization to crop growth promotion. However, several critical issues remain prominent in the current application of PSMs, including low field colonization efficiency, strain functional attenuation, unclear mechanisms of multi-strain synergy, and outdated fermentation processes, which severely constrain their large-scale implementation. Future endeavors should integrate cutting-edge technologies such as metagenomics, synthetic biology, and artificial intelligence-assisted design to directionally mine high-efficiency functional genes, construct intelligent synthetic microbial consortia, and optimize fermentation and application processes. These efforts will propel PSMs from the laboratory to the field, thereby providing critical technological support for efficient phosphorus recycling, reducing chemical fertilizer use while enhancing crop productivity, and fostering sustainable agricultural development. To address the above challenges, concerted efforts should be directed toward the following four priorities: (i) integrating cutting-edge technologies—including metagenomics, synthetic biology, and AI-assisted design—to mine efficient functional genes and construct intelligent synthetic consortia, thereby shifting the research paradigm from “single strain–single trait” to rational design of multi-strain consortia; (ii) incorporating PSM-based biofertilizers into policy frameworks for chemical fertilizer reduction, establishing product standards and registration/certification systems, and lowering application barriers through targeted subsidies and agricultural extension training; (iii) overcoming bottlenecks in shelf life and formulation stability of microbial inoculants, and developing “strain–scenario matching” intelligent recommendation systems; and (iv) prioritizing PSM promotion under suitable conditions such as alkaline sandy loam soils, with inoculation strategies determined in conjunction with soil testing to avoid indiscriminate application. Future efforts should focus on optimizing fermentation and application techniques and strengthening field validation, so as to facilitate the translation of PSMs from laboratory to field, and to provide critical scientific and technological support for efficient phosphorus cycling, chemical fertilizer reduction and efficacy enhancement, and sustainable agricultural development.

## Figures and Tables

**Table 1:** Types of phosphate-solubilizing microorganisms.

Microbial Category	Typical Genera/Species	Source
Bacteria	*Pseudomonas moraviensis*, *Bacillus cereus*, *Bacillus aryabhattai*	[45]
	*Bacillus megaterium*, *Azotobacter chroococcum*, *Pseudomonas putida*	[54]
	*Bacillus velezensis* UFV 3918	[55]
	*Pediococcus pentosaceus* PSM16	[56]
	*Acinetobacter johnsonii*, *Pseudomonas glycinae*	[57]
	*Priestia megaterium*, *Bacillus subtilis*	[58]
	*Enterobacter cloacae*, *Bacillus cereus*, *Bacillus megaterium*	[59]
	*Enterobacter hormaechei*, *Bacillus atrophaeus*	[60]
	*Burkholderia sp.* HS5, *Pantoea* sp. CL37	[61]
	*Pseudomonas*, *Pantoea*, *Amycolatopsis*	[62]
	*Escherichia coli*	[63]
	*Rhizobium tropici* LNP6	[64]
	*Bacillus thuringiensis* HY-12, *Duganella* sp.	[65]
	*Enterobacter asburiae*	[66]
	*Enterobacter cancerogenus*, *Acinetobacter lwoffii*, *Brevundimonas vesicularis*, *Aeromonas caviae*	[67]
	*Klebsiella aerogenes*	[68]
	*Pantoea trifolii*, *Pantoea formicae*, *Bacillus velezensis*	[69]
	*Micrococcus* sp.	[70]
Fungi	*Apophysomyces spartima*	[71]
	*Cladosporium* sp. 1EM.P1, *Penicillium steckii*	[72]
	*Aspergillus flavus*, *Penicillium crustosum* C3, *Metarhizium robertsii*	[73]
	*Penicillium oxalicum*	[74]
	*Talaromyces pinophilus*	[75]
	*Talaromyces* sp. MC-F2	[76]
	*Candida*	[77]
	*Aspergillus niger*	[78]
	*Pleurotus ostreatus*, *Rhizoctonia solani*, *Sclerotium rolfsii*	[79]
	*Sagenomella diversispora*, *Penicillium waksmanii*	[80]
	*Haematonectria ipomoeae*, *Eleutherascus lectardii*, *Pochonia chlamydosporia var. catenulata*, *Acremonium polychromum*	[81]
	*Aspergillus tubingensis*	[82]
	*Funneliformis mosseae*	[71]
	*Glomus multisubtensum*, *Rhizophagus intraradices*	[83]
	*Trichoderma* spp.	[48]
Actinomycetes	*Streptomyces*	[1]
	*Streptomyces cellostaticus*, *Streptomyces durhamensis Streptomyces griseoplanus*	[49] [50]

**Table 2:** Organic acids produced by PSMs.

PSMs	Organic Acids	Source
*Paraburkholderia fungorum* UFLA 04-21, *Pseudomonas anuradhapurensis* UFPI B5	citric acid, malic acid, gluconic acid	[110]
*Klebsiella* sp. M2, *Kluyvera* sp. M8	oxalic acid, ascorbic acid, citric acid, succinic acid	[111]
*Aspergillus tubingensis*	succinic acid, gluconic acid, oxalic acid, citric acid	[112]
*Aspergillus niger* H1	lactic acid	[113]
*Pseudomonas fluorescens*, *Pseudomonas poae*, *Pseudomonas trivialis*	gluconic acid, oxalic acid, 2-ketogluconic acid, lactic acid, succinic acid, formic acid, citric acid, malic acid	[114]
*Acinetobacter calcoaceticus* P1, *Acinetobacter pittii* P2	acetic acid, propionic acid	[100]
*Funneliformis mosseae*, *Bacillus megaterium*	malic acid, oxalic acid, acetic acid	[101]
*Enterobacter* sp.	citric acid	[115]
*Enterobacter* sp. 15S	citric acid, fumaric acid, alpha-ketoglutaric acid, malic acid, oxalic acid	[116]
*Pseudomonas aeruginosa* AAC1	succinic acid, gluconic acid	[117]

## Data Availability

No new data were created or analyzed in this study. Data sharing is not applicable to this article.
